# Rapid Polyp Classification in Colonoscopy Using Textural and Convolutional Features

**DOI:** 10.3390/healthcare10081494

**Published:** 2022-08-08

**Authors:** Chung-Ming Lo, Yu-Hsuan Yeh, Jui-Hsiang Tang, Chun-Chao Chang, Hsing-Jung Yeh

**Affiliations:** 1 Graduate Institute of Biomedical Informatics, College of Medical Science and Technology, Taipei Medical University, Taipei 110301, Taiwan; 2 Graduate Institute of Library, Information and Archival Studies, National Chengchi University, Taipei 116011, Taiwan; 3 Division of Gastroenterology and Hepatology, Department of Internal Medicine, School of Medicine, College of Medicine, Taipei Medical University, Taipei 110301, Taiwan; 4 Division of Gastroenterology and Hepatology, Department of Internal Medicine, Taipei Medical University Hospital, Taipei 110301, Taiwan; 5 Research Center for Digestive Medicine, Taipei Medical University, Taipei 110301, Taiwan

**Keywords:** colorectal cancer, colon polyp, image features, convolutional neural network

## Abstract

Colorectal cancer is the leading cause of cancer-associated morbidity and mortality worldwide. One of the causes of developing colorectal cancer is untreated colon adenomatous polyps. Clinically, polyps are detected in colonoscopy and the malignancies are determined according to the biopsy. To provide a quick and objective assessment to gastroenterologists, this study proposed a quantitative polyp classification via various image features in colonoscopy. The collected image database was composed of 1991 images including 1053 hyperplastic polyps and 938 adenomatous polyps and adenocarcinomas. From each image, textural features were extracted and combined in machine learning classifiers and machine-generated features were automatically selected in deep convolutional neural networks (DCNN). The DCNNs included AlexNet, Inception-V3, ResNet-101, and DenseNet-201. AlexNet trained from scratch achieved the best performance of 96.4% accuracy which is better than transfer learning and textural features. Using the prediction models, the malignancy level of polyps can be evaluated during a colonoscopy to provide a rapid treatment plan.

## 1. Introduction

Colorectal cancer (CRC) is the fourth most common newly diagnosed internal cancer in the United States [1]. In 2020, a total of 147,951 new CRC cases and 52,300 CRC-related deaths were reported [1] including gastrointestinal (GI)-related mortality [2]. The risk factors are drinking, consuming red meat or processed meat, sedentary lifestyle, overweight, smoking, and genetic diseases [3,4]. However, genetic problems are less than five percent associated with colorectal cancer [4,5]. The possible symptoms are blood in the stool, changes in bowel habits, weight loss, anemia, palpable mass, tenesmus, abdominal pain, and fatigue. A CRC often transforms from a benign polyp to a malignant one [6] and can be diagnosed by biopsy-proven tissues obtained from colonoscopy.

Polyp types can be divided into non-neoplastic (hyperplastic polyp) and neoplastic polyps (adenomatous polyps) [7,8]. Hyperplastic polyps are usually <1 cm in diameter and may occur in any part of the colon. They are not considered cancerous unless they are sufficiently large to cause complications, and regular examination is recommended in most cases. Adenomatous polyps including adenomas and sessile serrated adenomas (SSA) are important precursors to the majority of colorectal cancer. Adenomas can be classified into tubular adenomas, tubular villous adenomas, and villous adenomas according to pathological classification. Other rare polyp types are hamartoma, pseudopolyps, carcinoid tumors, and connective tissue polyps. About 70% of colorectal cancers originate from adenomatous polyps. In contrast, 25–30% of colorectal cancer cases originate from sessile serrated polyps [9]. If colon adenomas are removed by colonoscopic polypectomy, patient mortality is reduced by 53% [10]. Consequently, detecting early colon adenomatous polyps is critical. In addition, for patients receiving anticoagulants or antiplatelet drugs such as warfarin and clopidogrel, immediate polypectomy is not recommended [11]. The colonoscopy examination simultaneously provides more information of polyp location and surrounding tissues for further treatment.

With the advancements in image processing and machine learning techniques, computer-aided diagnosis (CAD) systems have been proposed to assist clinical endoscopists to identify different polyp types. In the past literature, CAD has been used by radiologists to detect colon polyps in computed tomography colonoscopy [12,13,14]. Additionally, the automatized colon polyp segmentation was proposed [15]. Recently, deep convolutional neural networks (DCNN) have been proposed for colon polyp detection [16,17], segmentation [15,18], and classification [19]. The key point of detection task is rapid. The DCNN used in the studies can detect polys in real-time such as YOLO algorithms [16]. To correctly analyze the polyp tissues, segmentation DCNN including Focus U-Net was introduced for a better region extraction. As a CAD used in malignancy evaluation, handcrafted and DCNN features were proposed to classify polyps in endoscopy video-frames [19]. As a widely used artificial intelligence technique, DCNN has been used in the various applications in colonoscopy. For polyp classification, both handcrafted and DCNN features are useful. However, more complete comparisons should be established to realize the differences between features, networks, and training methods.

**Figure 1:**
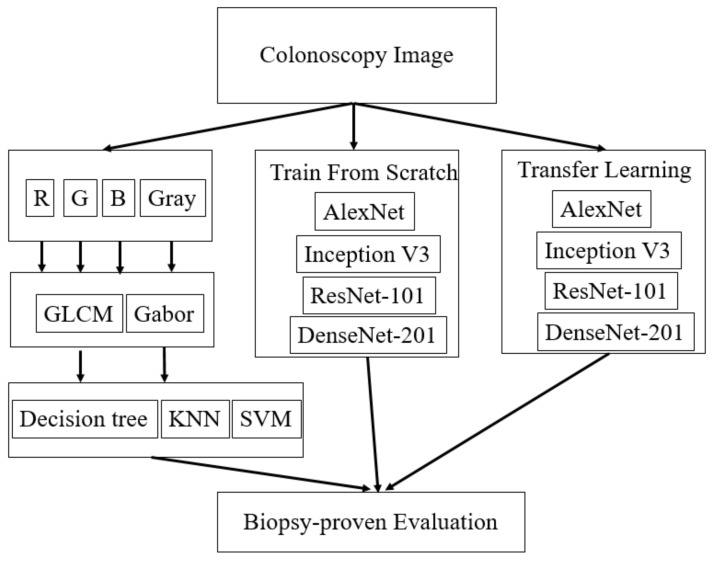
The flowchart of the polyp classification using colonoscopy image features.

To explore the classification ability and practice in clinical diagnosis, this study proposes using CAD systems for polyp classification in colorectal endoscopic images using different features, networks, and training methods. As shown in Figure 1, various approaches were implemented to compare the performance differences between machine learning using texture features and deep learning using DCNN features, the performance differences between DCNN models trained from scratch and transfer learning, and the performance differences between various DCNN architectures. The evaluations would provide more practical advice to gastroenterologists about using CAD for polyp classification in colonoscopy.

## 2. Materials and Methods

### 2.1. Colonoscopy Images

**Figure 2:**
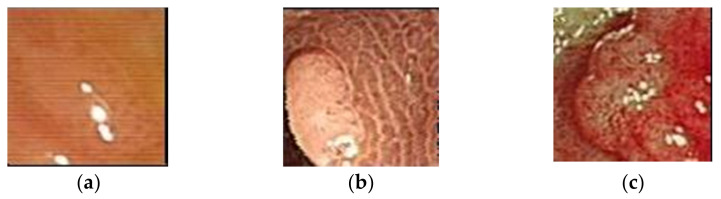
Different polys in endoscopy: (**a**) hyperplastic polyps; (**b**) adenoma; (**c**) adenocarcinoma.

This study was approved by the Taipei Medical University-Joint Institutional Review Board (approval no. N201802090c) on 25 February 2020. Between 1 January 2018 and 27 July 2018, 1991 patients underwent colonoscopy. Among these patients, 1053 were biopsy-proven to have hyperplastic polyps, 732 had adenomas, and 206 had adenocarcinomas. The collected colonoscopic images were obtained from colonoscopes (GF-260 and 290, Olympus Corporation, Tokyo, Japan). The format is jpeg with the resolution of 640 × 480. A total of 24 bits were used for a pixel, that is, the bit depth is 8 for red (R), green (G), and blue (B) individually. In the experiment, patient information was removed and the image part completely presenting the lesion area was cropped to be the image database (Figure 2). Colon polyps usually have a round or oval shape under the colonoscopy.

### 2.2. Textural Features

Quantitative image analysis is widely used for medical images [20,21]. Some image features used to interpret lesions can be observed by human eyes such as color and shape. Other types of image features, such as texture features, are subtle and denote correlations between adjacent pixel values. From the visual observations by gastroenterologists, the lesions including hyperplastic polyps, adenomas, and adenocarcinomas as shown in Figure 1 are mass-like which is related to shape properties. However, the light reflection and color differences would cause the lesion segmentation to fail. That is, shape features are hardly well-extracted from poor segmentations. Alternatively, texture features have been proposed in many CAD systems. The pattern differences between various lesion types can provide meaningful diagnostic information including the light reflection appeared in endoscopy. To present the color difference among different types, the texture features can be extracted from different color channels individually. Thus, texture features including gray-level co-occurrence matrix (GLCM) [13] and Gabor features [22,23] were proposed in this study for polyp classification.

GLCM extracts the spatial correlations between pixels as the texture features. First, the co-occurrence matrices *p* = [p (i, j|d, θ)] are generated to show the frequencies of each pixel (a gray value i) and its neighboring pixels (a gray value j) between a distance d and the direction θ. In the experiment, one pixel distance and four directions: 0°, 45°, 90°, and 135° were calculated and averaged. From the matrices, the statistical analysis was performed to generate various GLCM features including energy, mean, entropy, variance, correlation, homogeneity, dissimilarity, angular second moment, and contrast [24]. These features present the value distributions of tissue patterns. Energy is the sum of the squares of the element values in GLCM. If all values in the matrix are equal, the energy value is small; conversely, if some of the values are large and others are small, the energy value is large. A large energy value indicates a more uniform and regularly changing texture pattern. Entropy expresses the randomness of the texture. It is a measure of the amount of information that the image has, such as uniformity or complexity. When all pixels in the matrix are almost equal, entropy is relatively large. Contrast reflects the distribution of values in the matrix. The greater the grayscale difference, the greater the contrast and the greater this value. Correlation reflects the similarity among pixels in the matrix in a row or column. Homogeneity can also be called variance, which reflects the homogeneity of the image texture. If the image texture is uniform between different areas and changes slowly, homogeneity will be greater, and if the image texture is nonuniform, homogeneity will be smaller.

**Figure 3:**
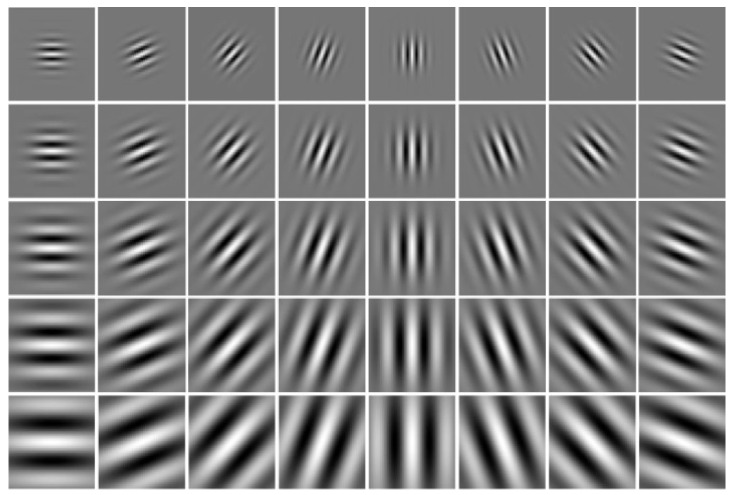
The 40 Gaussian filters in the Gabor filter.

**Figure 4:**
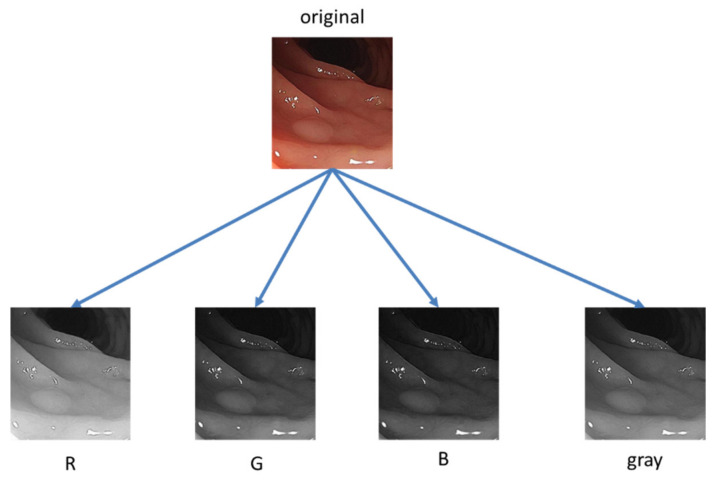
Conversions from a RGB image to four R, G, B, and grayscale images.

Gabor wavelets generated another kind of texture feature used in the experiment which was performed after Fourier transform. The Gabor features with various scales and rotations were then created. A total of forty Gabor filters in five scales and eight orientations are shown in Figure 3. In the Gabor, the sinusoid frequency and the orientation of the normal to the parallel stripes are used [25]. Gabor filter is used for extracting texture patterns such as what kind of specific frequency appeared in the pixels. The Gabor filter has real and imaginary parts that are orthogonal to each other. The two can form a complex number or be used alone. After filtering the real part, the image will be smooth, and filtering the imaginary part is used to detect edges [22]. Texture features are extracted from gray-scale pixels. Thus, from the original color image, three color channels were separated into three images. Additionally, a transformed gray-scale image was generated. As shown in Figure 4, four images were used for the feature extraction.

The use of GLCM and Gabor textures refer to the complete domain information, that is, Gabor collected texture features from frequency domain and GLCM collected texture features from spatial domain. After feature extraction, these image features were combined in various classifiers to establish polyp classification models. A total of 21 classifiers from MATLAB Classification Learner App (MathWorks Inc., Natick, MA, USA) were used, including decision tree, logistic regression, k-nearest neighbors, ensemble learning, and support vector machine (SVM). A 10-fold cross-validation was also performed during model evaluation. Principal component analysis was also performed as the feature selection to deal with the numerous features. The analysis reduces the feature dimension but minimizes information loss at the same time [26].

### 2.3. DCNN Features

**Figure 5:**
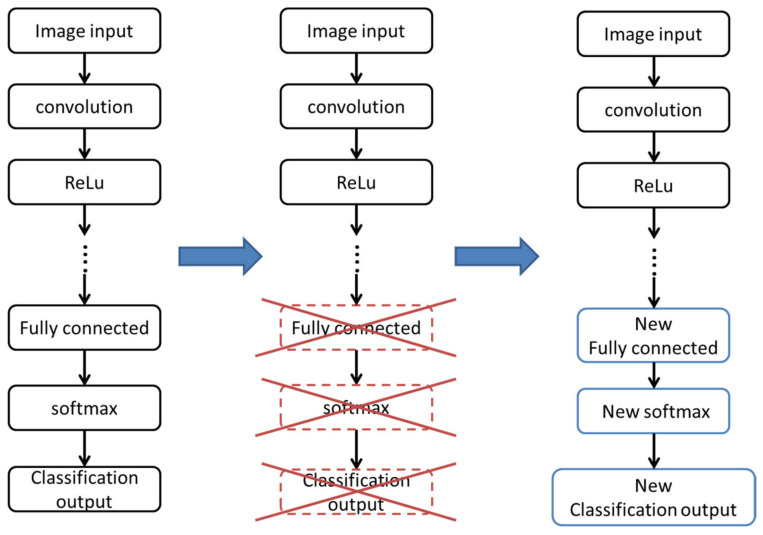
Illustration of transferred convolutional neural network.

DCNN is a deep learning technique that uses multiple layers in artificial neural networks [27,28,29]. Image features can be automatically extracted through linear or nonlinear transformation in multiple processing layers [30]. DCNN does not require the quantification of features through artificially designed metrics [31]. The essential architecture is composed of convolution layers, pooling layers, fully connective layers, and activation (nonlinearity) layers. The success of DCNN is based on the statistical analysis used to generate feature rules for the following classification. Therefore, a large number of input images is necessary. However, in the medical field, image data are not easily obtained such as natural images. To solve this problem, transfer learning was introduced to use features obtained from a pretrained model [32]. This is also called knowledge transfer, which means acquiring the knowledge of how to perform pattern recognition in natural images and using it in medical image classification. At present, the most widely known image database for transfer learning is ImageNet. In its implementation, the last few layers were removed from the pretrained model and were replaced with new layers. Then, the polyp images were fed to train parameters of new layers. An illustration is shown in Figure 5.

In the experiment, two ways were used to train a DCNN model. A DCNN trained from scratch means all the parameters for feature extraction and classification are learned from the target image database, i.e., the colonoscopy in this study. Another way to train a model is transferring parameters from a pre-trained big dataset such as ImageNet. However, ImageNet does not have too many colonoscopy images and may not be as helpful as expected. Thus, the comparisons are shown in this study to emphasize the differences. Moreover, the performances of different DCNN architectures were compared including AlexNet [33], Inception-V3 [34], ResNet-101 [35], and DenseNet-201 [36]. In the model training, the training and test datasets were randomly selected. Each network was trained 10 times, and the averaged accuracy values were regarded as the final result.

## 3. Results

In the experiment, input images were firstly divided into R, G, B, and grayscale images. After extracting GLCM and Gabor features, 21 classifiers were used. That is, the results contained 4 image types × 2 feature types × 21 classifiers = 168 prediction models with 10-fold cross-validation. The highest accuracy of 75.6% was obtained using GLCM from B images (Table 1), and the area under receiver operating characteristic curve was 0.82.

Using DCNN features, the performances of four types of DCNN with and without transfer learning were also explored, including AlexNet, Inception-V3, Resnet-101, and DenseNet-201. The parameters used in the training are learning rate = 0.001 and mini batch size = 64 to gradually achieve the local minimum with affordable image number. Epoch as the training iteration is set 30 for train from scratch and 3~15 for transfer learning. The determination is based on when to achieve the training convergence.

In Table 2, without transfer learning, the networks achieved the accuracies of 96.4%, 82.4%, 80.6%, and 87.4%, respectively. All of them have accuracy higher than 80% and the best one is 96.4%. Considering transfer learning in Table 3, the accuracies were 81.3%, 78.2%, 85.3%, and 87.7%. Inception-V3 only had 78.2% but still better than conventional texture features. The best one is 87.7% which is no better than AlexNet trained from scratch.

## 4. Discussion

CAD has been used for polyp detection under computed tomography [13]. Based on the success, this study explored extracting image features from colonoscopy image for polyp type classification. Recent literature proposed using texture features only or using DCNN features only in the classification of colon polyps [29,37]. The implementation of texture features is time-consuming, while it relatively costs less than DCNN. Nevertheless, texture features are easier to be explained. In the training of DCNN, a large image dataset and computational power are required. Although it may generate a higher accuracy, not all the medical intuitions can afford the computation power. With respect to achieving a higher accuracy, DCNN trained from scratch and transfer learning were implemented. According to the result, AlexNet trained from scratch can achieve the best performance of 96.4% accuracy. Transfer learning may not improve the performance with respect to the different networks used for the colonoscopy images. Some performances were increased or decreased or similar. Nevertheless, the worst one had 78.2% accuracy which was still higher than the best texture features, i.e., 75.6%.

Compared to a recent study using handcrafted and DCNN features for polyp classification [19], Ay, Betul et al. obtained 96.3% to 98.3% accuracies from different combinations of features and classifiers using video-frames of 80 participants. Although the number of 80 patients is much smaller than the 1991 patients used in this study, DCNN features performed better than handcrafted features in both studies. The accuracies higher than 96% would show the classification abilities of DCNN in polyp classification.

In clinical use, training from scratch may take more time compared to transfer learning. However, the accuracy difference is substantial such as 15.1% between AlexNet trained from scratch and transfer learning. It seems necessary to train an optimal model for a specific target task to obtain a good performance. Another way to improve the performance would be combining various features such as texture features or intensity features and various DCNN features in machine learning classifiers. This relates to more techniques about feature combination and feature selection. Whether the model can be applied to other datasets generated in different settings would be the next experiment. Then, we can estimate if a customized model is needed for different datasets and training methods. Meanwhile, a split validation would be performed to obtain more comparable results [38]. So far, the result shows that the prediction model can help gastroenterologists determine the polyp types during a colonoscopy.

It is helpful for gastrologists to predict the possible pathological results of polyps. With the prediction model based on the image features, gastrologist can have an early estimation of tissue malignancy. Some treatment plans can be arranged in advance without having to wait a few weeks. Using an image-based estimation model on other modalities is also helpful, including abnormal detections in capsule endoscopy which would be a time-consuming task for gastrologist. More trial or experiments will be executed after the preparation of data collection.

Compared with the general population, inflammatory bowel disease has a higher incidence of colorectal polyps [39] and colon cancer [40,41]. The proposed method may be used to predict the severity of intestinal mucosal pathological outcomes of inflammatory bowel disease in the future.

## 5. Conclusions

This study proposed the CAD system for the classification of polyp types using colonoscopy. Various features, networks, and training methods were implemented in the experiment. GLCM texture features in the B channel had the accuracy of 75.6%, while AlexNet trained from scratch obtained the accuracy of 96.4%. Based on the performance comparisons, DCNN can achieve a substantial performance and training from scratch is a promising way to build a model if the image data are good enough. The evaluations would provide more practical advice to gastroenterologists about using CAD for polyp classification during a colonoscopy. More CAD systems for intestinal tumors or inflammatory bowel diseases such as Crohn’s disease and ulcerative colitis would be possible in the future.

## Figures and Tables

**Table 1:** The top five accuracies using the texture features and different classifiers.

Model Type	Accuracy	Feature
Ensemble Bagged Trees	75.6%	GLCM_B
Coarse KNN	75.0%	GLCM_B
Ensemble Booted Trees	73.9%	GLCM_G
Ensemble RUSBooted Trees	73.5%	Gabor_B
Quadratic SVM	72.8%	GLCM_B

B = blue channel; G = green channel.

**Table 2:** The performances of various convolutional neural networks trained from scratch.

Train from Scratch	Accuracy	Sensitivity	Specificity
Alex	96.4%	95.7%	97.2%
Inception-V3	82.4%	78.7%	85.9%
ResNet-101	80.6%	87.2%	74.5%
DenseNet-201	87.4%	86.2%	87.7%

**Table 3:** The performances of various convolutional neural networks using transfer learning.

Transfer Learning	Accuracy	Sensitivity	Specificity
Alex	81.3%	90.4%	72.6%
Inception-V3	78.2%	67.0%	87.7%
ResNet-101	85.3%	81.9%	87.7%
DenseNet-201	87.7%	83.0%	91.5%
